# 

**Published:** 1917-10-06

**Authors:** 


The Modern Newspaper of Administrative Medicine and Institutional Life.

Telegraphic Address: OSPEDALE, RAND, LONDON. Telephone Number: 2734 GERRARD.

No. 1635, Vol. LXIII. | Saturday, October 6th, 1917. | PRICE ONE PENNY

